# Red cell microparticles produced using high-pressure extrusion enhance both primary and secondary hemostasis

**DOI:** 10.1007/s43440-024-00688-0

**Published:** 2025-01-08

**Authors:** Snigdha Sama, Sunjoo Cho, Ashish K. Rehni, Wenche Jy, Kunjan R. Dave

**Affiliations:** 1 Department of Neurology, Peritz Scheinberg Cerebral Vascular Disease Research Laboratories, University of Miami Miller School of Medicine, 1600 NW 10th Ave RMSB #7046, Miami, FL 33136 USA; 2 Neuroscience Program, University of Miami Miller School of Medicine, Miami, FL 33136 USA; 3 The Wallace H. Coulter Platelet Laboratory, Division of Hematology/Oncology, Department of Medicine, University of Miami Miller School of Medicine, Miami, FL USA

**Keywords:** Hemostatics, extracellular vesicles, hemostasis, platelet aggregation

## Abstract

**Background:**

Current therapies to treat excessive bleeding are associated with significant complications, which may outweigh their benefits. Red blood cell-derived microparticles (RMPs) are a promising hemostatic agent. Previous studies demonstrated that they reduce bleeding in animal models, correct coagulation defects in patient blood, and have an excellent safety profile. However, their exact mechanism of action is not known. We investigated the potential role of RMPs on primary and secondary hemostasis.

**Methods:**

To evaluate the effects of RMPs, prepared using high-pressure extrusion, on primary hemostasis, we employed platelet aggregometry with platelet inhibitors, eptifibatide, and ticagrelor, with and without RMPs. To evaluate their effects on secondary hemostasis, we employed thromboelastography with plasma deficient in factors VII, VIII, IX, XI, and XII with and without RMPs.

**Results:**

We found that RMPs significantly increased collagen-induced platelet aggregation. However, there were no significant differences with and without RMP in the presence of the platelet inhibitors, indicating that RMPs may work through these receptors, either directly or indirectly. For secondary hemostasis, RMPs significantly decreased clotting times for plasma deficient in factors VII, VIII, IX, and XI but not in XII.

**Conclusions:**

Our results indicate that RMPs enhance primary hemostasis and both pathways of secondary hemostasis.

**Supplementary Information:**

The online version contains supplementary material available at 10.1007/s43440-024-00688-0.

## Introduction

Excessive bleeding due to traumatic injury is one of the leading causes of death in the world [1]. Currently, several therapies such as transfusion, antifibrinolytics, and blood factors are used to treat hemorrhage [2]. However, these therapies have major limitations. Transfusions are associated with complications such as hemolytic reactions, infectious disease transmission, and transfusion-related acute lung injury [2]. Studies looking at antifibrinolytics such as tranexamic acid have had conflicting results, with some showing increased mortality [1, 3, 4]. Blood factors such as activated recombinant factor VII are associated with increased thromboembolic events [2]. An alternative hemostatic agent with benefits exceeding its side effects is needed.

Cell-derived microparticles (MPs) are one potential option. MPs are small membrane vesicles derived from platelets, endothelial cells, leukocytes, and red blood cells (RBCs) that expose phosphatidylserine, a phospholipid on the lipid membrane that can bind to different coagulation factors to enhance the coagulation cascade [5–7]. While the International Society of Extracellular Vesicles refers to MPs as extracellular vesicles, we refer to them here as MPs for consistency with our earlier publications [5, 8–10]. MPs derived from platelets, endothelial cells, and leukocytes contain tissue factor (TF), which, in excess, can trigger thrombotic disorders [11, 12]. On the other hand, TF was not detectable in RBC-derived MPs (RMPs) [6, 12–14]. Due to this, RMPs show the most potential of the MPs to be used as a hemostatic therapy.

Earlier studies have shown the benefits of RMPs as a hemostatic agent. In vitro studies found that RMPs enhanced platelet adhesion and platelet aggregation and initiated and enhanced thrombin generation [6, 12]. An ex vivo study found that RMP corrected thrombin generation time in blood samples from patients with various bleeding disorders [12]. In addition, several in vivo studies have found that RMP decreased bleeding. RMP reduced bleeding time in a rabbit model of busulfan-induced thrombocytopenia and in a rat model of Plavix-induced platelet dysfunction [12]. RMP also limited hematoma expansion and improved long-term outcomes in a rat model of collagenase-induced intracerebral hemorrhage [10]. In addition, RMP treatment resulted in significantly decreased hematomas in several comorbid conditions of intracerebral hemorrhage [15, 16]. Moreover, RMP did not produce thrombosis in the brain, heart, or lungs when administered at high doses in rats [9]. Taken together, these earlier studies demonstrate that RMPs are not only safe but also effective at enhancing hemostasis.

Based on these studies, the hemostatic action of RMPs has been shown to involve both the primary and secondary pathways of hemostasis [12]. However, this exact mechanism is not known. The mechanism of action of a drug may be critical to determine its optimal dosing, understand its side effects, and predetermine its interaction with other drugs [17, 18]. The goal of the current study is to evaluate the potential impact of RMPs on hemostasis. Specifically, we employed platelet inhibitors and factor-deficient plasma to evaluate how RMPs may enhance primary and secondary hemostasis, respectively.

## Materials and methods

### Preparation of RMP

RMPs were produced using type O + leukoreduced RBCs and high-pressure extrusion as previously described [12]. In brief, packed RBCs (OneBlood, Ft. Lauderdale, FL, USA) were washed twice with an equal volume of 0.9% saline by centrifugation at 650 × g for 10 min. RBCs were resuspended in an equal volume of saline and passed through the TS2 Bench-top Cell Disruptor (Constant Systems, Northants, UK) twice to produce RMPs. RMPs were washed twice with saline by centrifugation at 25,000 × g for 30 min. The resulting pellet was resuspended in saline containing thimerosal (50 µg/mL, final concentration), briefly sonicated, aliquoted into small vials, and stored at -80 °C. The number of RMPs was determined by counting CD235a-positive particles using a flow cytometer (additional information regarding flow cytometry can be found in the reference) (Figure S1) [5]. The procoagulant activity of the RMP was confirmed with a significant reduction in R-time in thromboelastography (TEG) using diluted platelet-poor plasma (PPP) (OneBlood) compared to saline.

### Thromboelastography (TEG)

The effect of RMPs on secondary hemostasis was evaluated using TEG (Haemonetics, Braintree, MA, USA) [12]. For this experiment, PPP (pooled plasma from 53 healthy donors) (George King Biomedical, Overland Park, KS, USA) and plasma deficient in factors VII, VIII, IX, XI, and XII (George King Biomedical) were used. Each factor-deficient plasma was mixed with PPP to produce 85%, 92.5%, and 96.25% factor-deficient plasma, which were incubated with either RMP (2.8 × 10^8^ particles/mL, final concentration) or saline (control) for 2 min at room temperature. This mixture (340 µL) was added to a 37 °C TEG cup preloaded with 20 µL of 0.2 M CaCl_2_ to start the coagulation. The test was run until lag time to initial fibrin formation (R-time, min), time to 20 mm amplitude from start of clot formation (K, min), velocity of clot formation (A, degree), and maximum strength of clot (MA, mm) were all recorded. Furthermore, the coagulation index (CI), a composite of the five parameters, was calculated using the following formula: CI = − 0.2454R + 0.0184 K + 0.1655MA – 0.0241 A – 5.022 [19]. A higher CI value indicates an increased procoagulant state and a lower CI value indicates decreased coagulant activity. All tests were done in triplicate.

### Blood collection and platelet aggregometry

This study was approved by the University of Miami Institutional Review Board (IRB ID: 20190814; effective date: 9/11/2019). All participants were self-reported healthy adults not taking any medication and gave voluntary and written informed consent. Blood was collected from donors (*n* = 7) in sodium heparin tubes. The subjects (4 males, 3 females) had an average age of 36.3 ± 4.1 years (range: 20–48 years). Blood was centrifuged at 200 × g for 10 min, and platelet-rich plasma (PRP) was separated. Platelets were counted using a hemocytometer. Based on this count, PRP was diluted with saline to obtain a platelet concentration of 250 × 10^6^ platelets/mL. Samples were analyzed using the platelet aggregometer within 6 h of blood draw.

The effect of RMPs on primary hemostasis was evaluated with a multi-channel platelet aggregometer (Chrono-Log, Havertown, PA, USA) using the turbidimetric method [12]. PPP (300 µL) was prepared for each sample by centrifuging diluted PRP at 14,000 × g for 3 min at room temperature to serve as a blank. PRP was incubated with either saline or the following inhibitors for 5 min at 37 °C in a glass cuvette with a magnetic stir bar: glycoprotein IIb/IIIa (GPIIb/IIIa) inhibitor, eptifibatide acetate (final concentration: 20 µg/mL), and adenosine diphosphate (ADP) P2Y_12_ receptor inhibitor, ticagrelor (final concentration: 12 µM). After baseline was confirmed to be stable for over a min, saline (control) or RMP (final concentration: 4 × 10^8^ particles/mL) was added to each mixture before the agonist. Aggregations were induced with the addition of collagen (final concentration: 16 ng/mL) and monitored for 20 min at 37 °C. The following groups were run in parallel: PRP + saline (control), PRP + RMP, PRP + inhibitor, and PRP + inhibitor + RMP. Data was recorded using the aggregometer software (Aggrolink 5, Chrono-Log), which calculated the area under the curve (AUC), slope, and amplitude. Results were calculated as percent of PRP + saline for each run and averaged together.

### Statistics

Data were analyzed using GraphPad Prism (Version 10, San Diego, CA). Using Grubbs’ test, we verified the absence of significant outliers. Two-way analysis of variance (ANOVA) with Fisher’s Least Significant Difference test for post-hoc analysis was used to evaluate the effect of RMP on platelet aggregation and secondary hemostasis. Student’s t-test was used to analyze the effect of collagen on RMP. A p-value < 0.05 was considered statistically significant, and all results are presented as mean ± standard error of the mean.

## Results

### Effect of RMP on blood coagulation

**Fig. 1:**
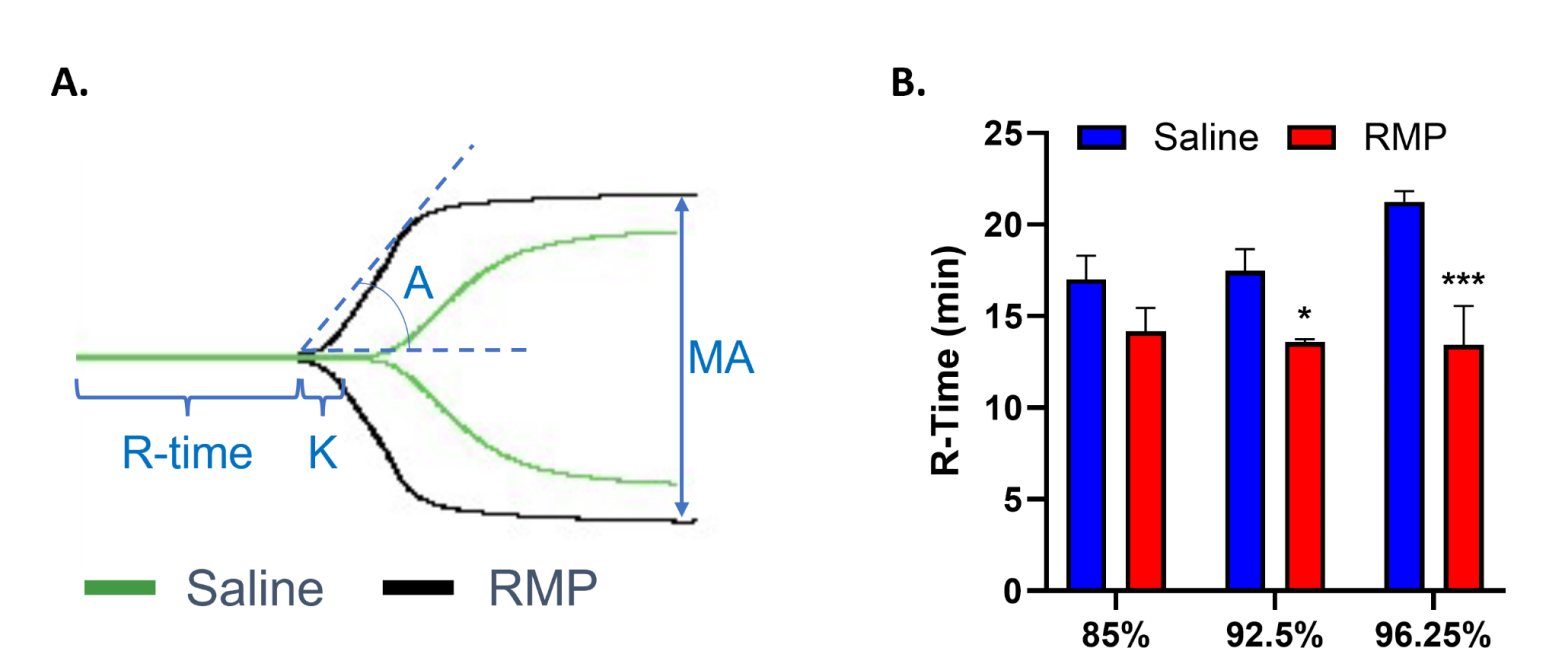
Effect of RMP on the extrinsic pathway. Saline or RMP was added to factor-deficient plasma, and R-time was evaluated as a measure of procoagulant activity using TEG. (A) Representative tracing from TEG. (B) RMP significantly enhances coagulation in factor VII deficiency (*n* = 3 for all) using two-way analysis of variance with Fisher’s Least Significant Difference. RMP: red blood cell-derived microparticles; R-time: lag time until initial fibrin formation; TEG: thromboelastography. *p < 0.05, and ***p < 0.001 vs. saline. Results are presented as mean ± standard error of the mean

To investigate the effect of RMP on the extrinsic pathway of secondary hemostasis, we focused on factor VII, its major component, using TEG (Fig. 1A). Two-way ANOVA analysis found RMP treatment had a significant effect (F_1,12_=22.06, *p* = 0.0005). RMP significantly decreased R-time in 92.5% (17.5 min to 13.6 min, *p* = 0.050) and 96.25% (21.2 min to 13.4 min, *p* = 0.009) deficient plasma compared to saline (*n* = 3 for all) (Fig. 1B). RMP decreased R-time in 85% deficient plasma (17.0 min to 14.2 min, *n* = 3), although this difference was not significant (*p* = 0.14).

**Fig. 2:**
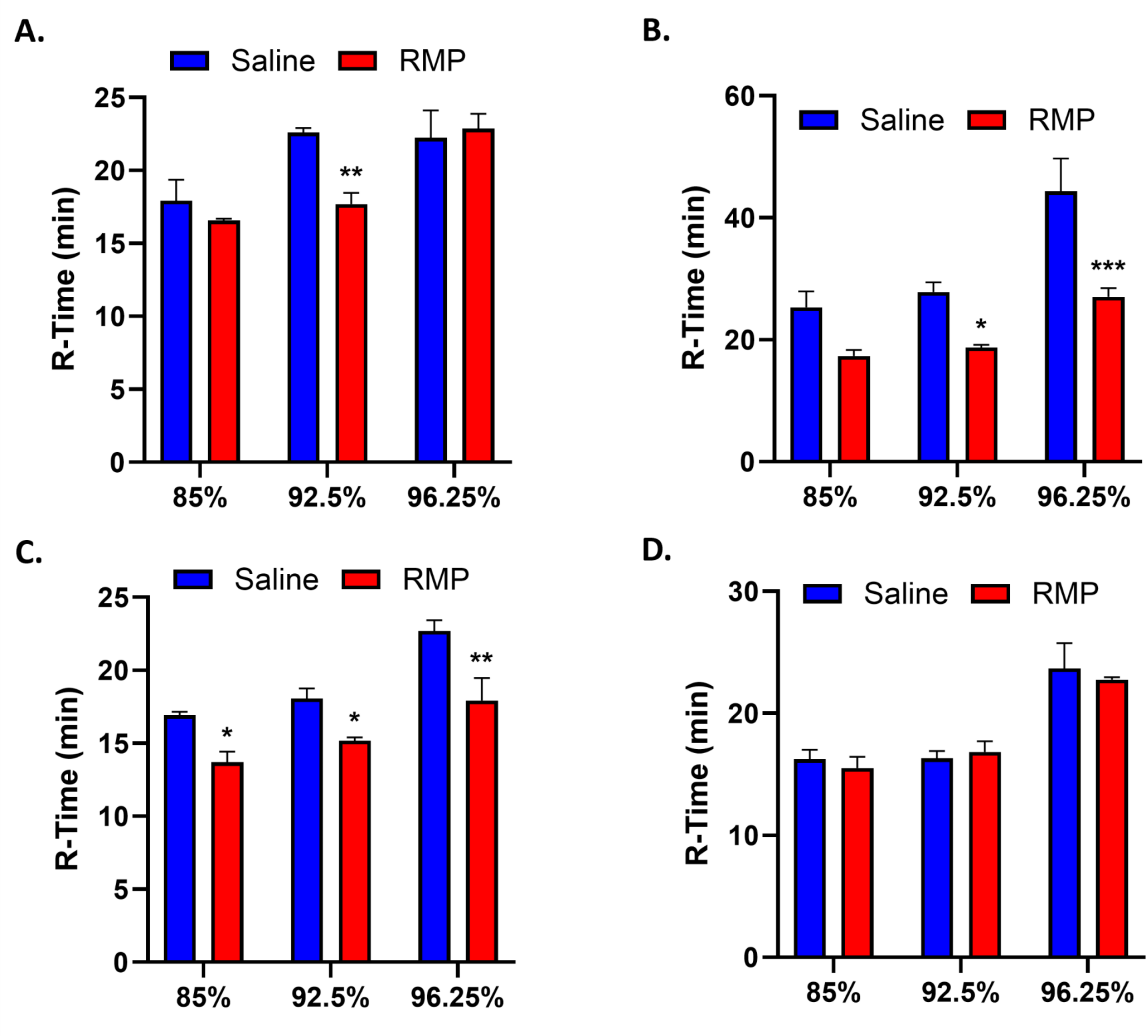
Effect of RMP on the intrinsic pathway. Saline or RMP was added to factor-deficient plasma, and R-time as evaluated as a measure of procoagulant activity using TEG. RMP significantly enhances coagulation in factor VIII (A), IX (B), and XI (C) deficiency but not in factor XII (D) deficiency (*n* = 3 for all) using two-way analysis of variance with Fisher’s Least Significant Difference. RMP: red blood cell-derived microparticles; R-time: lag time until initial fibrin formation; TEG: thromboelastography. *p < 0.05, **p < 0.01, and ***p < 0.001 vs. saline. Results are presented as mean ± standard error of the mean

To investigate the effect of RMP on the intrinsic pathway of secondary hemostasis, we focused on its major factors: VIII, IX, XI, and XII. For all factors tested, two-way ANOVA found a significant effect of factor deficiency (factor VIII: F_2,12_=11.54, *p* = 0.002; factor IX: F_2,12_=17.27, *p* = 0.0003; factor XI: F_2,12_=20.10, *p* = 0.0001; factor XII: F_2,12_=28.68, *p* < 0.0001). In addition, two-way ANOVA found a significant effect of RMP treatment for factor IX (F_2,12_=28.25, *p* = 0.0002) and factor XI (F_2,12_=29.57, *p* = 0.0002). For factor VIII, RMP significantly decreased R-time in 92.5% deficient plasma (22.6 min to 17.7 min, *p* = 0.009) compared to saline (*n* = 3 for both) (Fig. 2A). There was no significant decrease in 85% and 96.25% deficient plasma (*n* = 3 for both). For factor IX, RMP had a significant effect (*p* = 0.0002) and significantly reduced R-time in 92.5% (27.8 min to 18.7 min, *p* = 0.031) and 96.25% (44.3 min to 27 min, *p* = 0.0006) deficient plasma compared to saline (*n* = 3 for all) (Fig. 2B). RMP decreased R-time in 85% (25.3 min to 17.3 min), although this difference was not significant (*p* = 0.054). For factor XI, RMP had a significant effect (*p* = 0.0001) and significantly decreased R-time in 85% (16.9 min to 13.7 min, *p* = 0.016), 92.5% (18.1 min to 15.2 min, *p* = 0.028), and 96.25% (22.7 min to 17.9 min, *p* = 0.001) deficient plasma compared to saline (*n* = 3 for all) (Fig. 2C). For factor XII, RMP did not show any statistically significant effect on R-time in any of the factor-deficient plasma (*n* = 3 for all) (Fig. 2D). Results for K, A, MA, and CI for all factor-deficient plasma are presented in Table 1. Our results indicate that RMPs enhance secondary hemostasis through both the intrinsic and extrinsic pathways.

**Table 1:** TEG parameters of RMP on factor deficient plasma

Factor	Deficiency	K (min)		A (°)		MA (mm)		CI	
		-RMP	+RMP	-RMP	+RMP	-RMP	+RMP	-RMP	+RMP
**VII**	85%	5.4 ± 0.6	4.6 ± 0.3	35.2 ± 3.6	37.8 ± 2.4	37.0 ± 1.2	35.5 ± 0.3	-3.8 ± 0.1	-3.4 ± 0.2
	92.5%	4.6 ± 0.9	3.7 ± 0.9	38.3 ± 3.7	48.6 ± 5.8	37.5 ± 0.3	36.0 ± 0.7	-3.9 ± 0.2	-3.5 ± 0.2
	96.25%	5.2 ± 1.5	2.6 ± 0.5	35.7 ± 4.4	55.0 ± 5.4 (*p* = 0.009)	38.2 ± 0.6	40.0 ± 1.5	-4.7 ± 0.3	-3.0 ± 0.6 (*p* = 0.003)
**VIII**	85%	7.7 ± 0.1	6.1 ± 0.7	29.6 ± 2.5	22.7 ± 9.7	22.3 ± 0.6	21.8 ± 1.1	-6.3 ± 0.5	-6.0 ± 0.4
	92.5%	14.9 ± 4.8	10.3 ± 4.3	20.0 ± 3.6	28.4 ± 1.4	20.1 ± 0.4	20.2 ± 1.2	-7.5 ± 0.2	-6.6 ± 0.1
	96.25%	6.9 ± 0.8	5.2 ± 0.7	30.7 ± 2.1	33.8 ± 2.4	31.3 ± 1.4	31.7 ± 0.8	-5.9 ± 0.7	-6.1 ± 0.3
**IX**	85%	11.5 ± 1.3	5.5 ± 0.2	17.2 ± 1.3	30.1 ± 1.5 (*p* = 0.026)	25.4 ± 1.6	30.5 ± 0.0	-7.9 ± 0.4	-4.8 ± 0.4 (*p* = 0.002)
	92.5%	13.2 ± 3.8	5.2 ± 0.6 (*p* = 0.022)	18.5 ± 3.7	32.8 ± 3.2 (*p* = 0.005)	26.0 ± 0.2	31.5 ± 1.6 (*p* = 0.016)	-7.8 ± 0.4	-5.1 ± 0.5 (*p* = 0.001)
	96.25%	12.8^#^	10.0 ± 0.9	10.7 ± 2.6	17.7 ± 2.9	20.2 ± 2.3	26.3 ± 0.6 (*p* = 0.010)	-9.3#	-7.5 ± 0.4 (*p* = 0.047)
**XI**	85%	7.6 ± 0.5	5.8 ± 1.1	26.9 ± 2.0	38.1 ± 7.3	24.8 ± 0.9	23.3 ± 1.1	-5.6 ± 0.1	-5.3 ± 0.2
	92.5%	4.1 ± 0.1	4.1 ± 0.9	38.8 ± 1.8	34.3 ± 5.4	30.0 ± 0.7	29.6 ± 1.0	-5.4 ± 0.2	-4.6 ± 0.1
	96.25%	6.6 ± 0.3	4.6 ± 0.6	29.5 ± 1.1	38.5 ± 4.6	30.7 ± 1.2	32.5 ± 3.0	-6.1 ± 0.1	-4.9 ± 0.7 (*p* = 0.014)
**XII**	85%	4.5 ± 0.5	6.1 ± 0.6	39.8 ± 3.4	29.2 ± 1.9 (*p* = 0.020)	29.9 ± 0.9	29.7 ± 0.3	-4.8 ± 0.2	-4.3 ± 0.0
	92.5%	4.7 ± 0.8	4.9 ± 0.2	38.3 ± 4.1	36.7 ± 1.9	27.4 ± 0.8	31.0 ± 1.1 (*p* = 0.003)	-5.5 ± 0.2	-5.0 ± 0.0
	96.25%	7.1 ± 0.7	6.0 ± 0.4	24.7 ± 1.1	29.7 ± 3.3	27.0 ± 0.3	28.2 ± 0.2	-6.8 ± 0.5	-6.5 ± 0.1

Saline or RMP was added to factor-deficient plasma, and the parameters K, A, MA, and CI were evaluated using TEG (*n* = 3 for all, except for those marked with #, which had *n* = 1 as MA did not reach > 20 mm). Analysis performed using one-way analysis of variance with Fisher’s Least Significant Difference test as post-hoc analysis. K: time to 20 mm amplitude from start of clot formation; A: velocity of clot formation; MA: maximum strength of clot; CI; coagulation index-composite of all five parameters; TEG: thromboelastography. Results are presented as mean ± standard error of the mean

### Effect of RMP on platelet aggregation

**Fig. 3:**
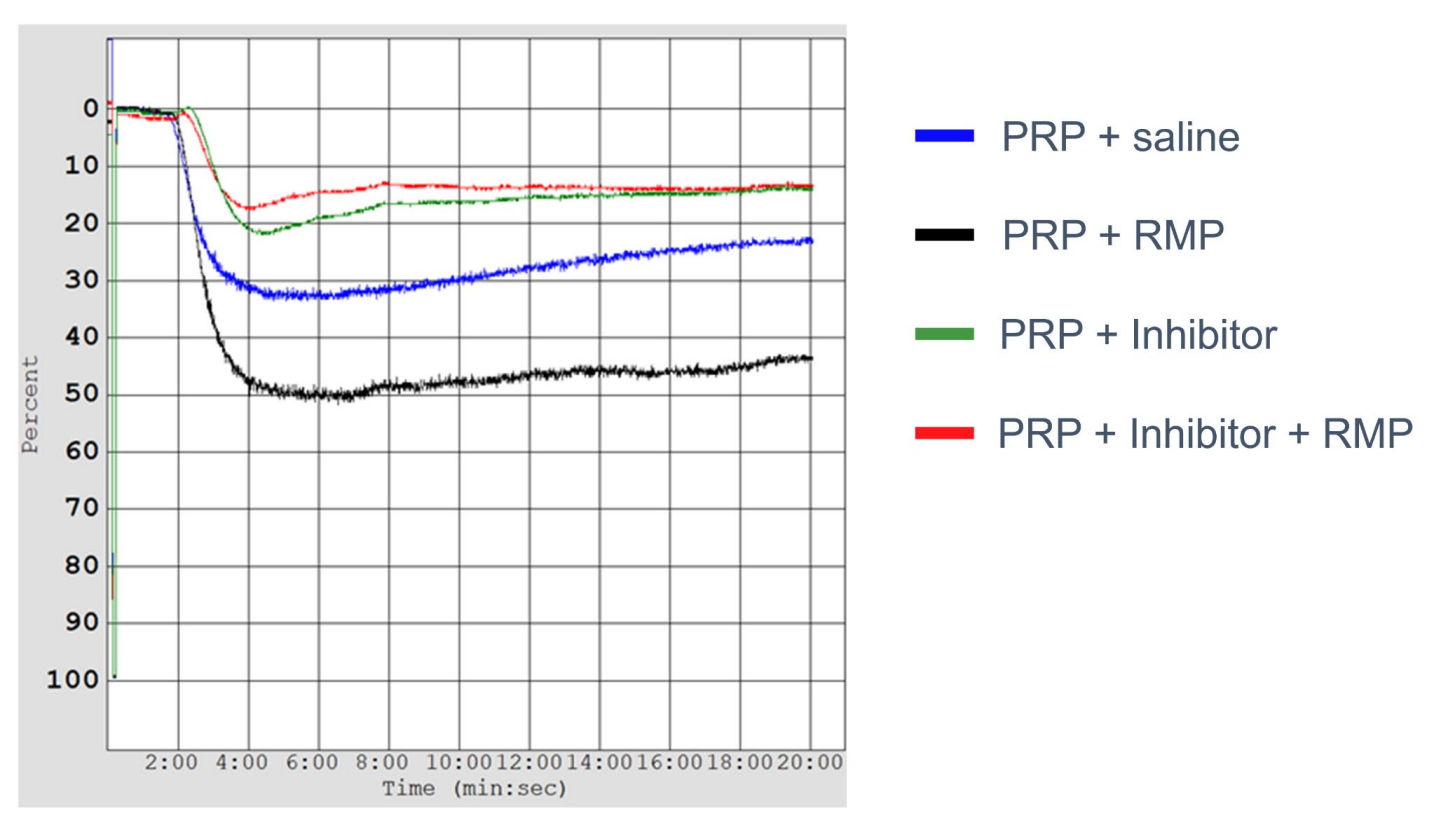
Representative tracing from platelet aggregometer. The effect of RMP on platelet aggregation was evaluated in the absence and presence of an inhibitor using a platelet aggregometer. PRP: platelet-rich plasma; RMP: red blood cell-derived microparticles

Previously, we found increased platelet aggregation in the presence of RMP when initiated by ADP and arachidonic acid [12]. In our current study, we found RMP significantly increased platelet aggregation following collagen-induced platelet activation (Fig. 3). AUC, slope, and amplitude increased by 35% (*p* = 0.035; t_6_ = 2.72), 32% (*p* = 0.032; t_6_ = 2.27), and 32% (*p* = 0.013; t_6_ = 2.96; *n* = 7 for all), respectively (Table 2).

**Table 2:** Effect of RMP on platelet aggregation

Effect of Collagen on RMP (*n* = 7)	AUC	Slope	Amplitude
**PRP + saline**	100	100	100
**PRP + RMP** (vs. PRP + saline)	135±13 *p* = 0.017	132±14 *p* = 0.032	132±10 *p* = 0.013
**Effect of Eptifibatide on RMP** (*n* = 7)	**AUC**	**Slope**	**Amplitude**
**PRP + saline**	100	100	100
**PRP + RMP** (vs. PRP + saline)	138±17 *p* = 0.013	142±22	139±15 *p* = 0.010
**PRP + eptifibatide** (vs. PRP + saline)	27±8 *p* < 0.0001	59±16	37±9 *p* = 0.0001
**PRP + eptifibatide + RMP** (vs. PRP + saline) (vs. PRP + RMP)	23±7 *p* < 0.0001 *p* < 0.0001	51±11 *p* = 0.026 *p* = 0.0002	37±9 *p* = 0.0001 *p* < 0.0001
**Effect of Ticagrelor on RMP** (*n* = 4)	**AUC**	**Slope**	**Amplitude**
**PRP + saline**	100	100	100
**PRP + RMP**	131±13	118±10	115±6
**PRP + ticagrelor**	66±15	70±16	72±13 *p* = 0.023
**PRP + ticagrelor + RMP** (vs. PRP + saline) (vs. PRP + RMP)	68±15 *p* = 0.004	77±9 *p* = 0.016	72±6 *p* = 0.025 *p* = 0.002

The effect of RMP on platelet aggregation was evaluated in the absence and presence of an inhibitor using a platelet aggregometer. Analyses performed using Student’s t-test (for the effect of collagen on RMP) and two-way analysis of variance with Fisher’s Least Significant Difference test as post-hoc analysis (for the effect of eptifibatide and ticagrelor on RMP). PRP: platelet-rich plasma; RMP: red blood cell-derived microparticles; AUC: area under the curve. Results are expressed as percent of PRP to account for individual differences and presented as mean ± standard error of the mean

As expected, both inhibitors suppressed platelet aggregation. Two-way ANOVA found that eptifibatide had a significant effect on AUC (F_1,24_=85.20, *p* < 0.0001), slope (F_1,24_=20.44, *p* = 0.0001), and amplitude (F_1,24_=70.99, *p* < 0.0001). Two-way ANOVA also found that ticagrelor had a significant effect on AUC (F_1,12_=14.70, *p* = 0.002), slope (F_1,12_=11.82, *p* = 0.005), and amplitude (F_1,12_=21.40, *p* = 0.0006).

In addition, we evaluated the effect of RMP in the presence of the inhibitors eptifibatide and ticagrelor. Two-way ANOVA found that eptifibatide × RMP treatment interaction was significant (F_1,24_=4.282, *p* = 0.050). In the presence of RMP and eptifibatide, the AUC is significantly decreased compared to the control (*p* < 0.0001) and PRP + RMP (*p* = 0.013), but not compared to the AUC of only eptifibatide (*n* = 7). In the presence of RMP and ticagrelor, the AUC is significantly decreased compared to the PRP + RMP (*p* = 0.004), but not compared to the control or only ticagrelor (*n* = 4). We found no significant difference in either inhibitor in the presence or absence of RMPs. The results for slope and amplitude are presented in Table 2 and demonstrate that although RMP can enhance platelet aggregation, this effect is lost in the presence of GPIIb/IIIa and P2Y_12_ receptor inhibitors, indicating that RMP may work through platelet activation.

## Discussion

Hemostatic agents with limited side effects are crucial to treat excessive bleeding. RMPs show promise, as highlighted in several animal models. Previously, RMPs were shown to enhance hemostasis [12]. Our study aimed to evaluate the potential impact of RMPs on primary and secondary hemostasis. We found that RMP significantly affected both the intrinsic and extrinsic pathways of secondary hemostasis; more specifically, RMPs enhanced procoagulant activity in the presence of low levels of factors VII, VIII, IX, and XI but not factor XII. In addition, RMPs significantly increased platelet aggregation in the presence of collagen, although not in the presence of GPIIb/IIIa or ADP P2Y12 receptor inhibitors.

Several studies have investigated the effect of RMPs with conflicting results. One study found that RMPs can initiate thrombin generation through a factor XII-dependent manner, while others found that thrombin generation was factor IX-dependent [6, 13, 20]. These studies used RMPs generated with calcium ionophore and storage induction, respectively. Previous studies have looked at the biological activities of RMPs generated through long-term storage and found increased pro-inflammatory effects [21]. As earlier studies found significant differences in physical properties, protein content, and lipid raft proteins in RMP generated using different methods, it is difficult to rely solely on these results to determine the phenotype of RMPs generated using high-pressure extrusion [22, 23].

Our laboratory previously found that RMPs generated with high-pressure extrusion enhance both pathways of secondary hemostasis [12]. Unlike prior studies which looked at secondary hemostasis factors activated by RMPs, we wanted to focus on factors enhanced by RMPs. Therefore, we used differing percentages of factor-deficient plasma rather than 100% factor-deficient plasma in our current study. For the extrinsic pathway, RMPs enhanced coagulation in factor VII-deficient plasma despite not having any detectable levels of TF [6, 12–14]. Although there is conflicting data on RMPs containing TF, most studies found RMPs contained no detectable levels of TF and several studies found that the procoagulant activity of RMPs is independent of TF [6, 12–14, 24–26].

For the intrinsic pathway, RMP had a significant effect on coagulation in factor IX- and XI-deficient plasma. Interestingly, RMP had an effect at 92.5% factor VIII-deficient plasma but not 96.25% factor VIII-deficient plasma. This may indicate that RMP are not able to compensate for a lack of factor VIII and a minimum concentration of factor VIII is needed for RMPs to work effectively. Additionally, RMP had no effect on factor XII-deficient plasma, which correspond with a prior study that found RMPs were not effective in initiating thrombin generation in factor XII-deficient plasma and that RBC membranes were able to activate factor IX [6, 27]. However, another interpretation is that a minimum concentration of factor XII is required for RMPs to have a significant effect.

Unlike secondary hemostasis, little research has been done on the effect of RMPs on primary hemostasis. Previously, our laboratory found that RMPs enhance primary hemostasis [12]. At moderate shear, RMPs resulted in ~ 20% larger platelet aggregate size and amplified and sustained platelet aggregation with the addition of arachidonic acid and ADP, respectively. In the current study, RMP significantly increased platelet aggregation with the addition of collagen. GPIIb/IIIa inhibitor, eptifibatide, and ADP P2Y_12_ receptor inhibitor, ticagrelor were used to investigate how RMPs affect primary hemostasis. GPIIb/IIIa is a critical component of platelet-platelet interactions, and ADP P2Y_12_ is essential for aggregation amplification and stabilization [28]. Platelet aggregation significantly decreased in the presence of the inhibitors and RMP compared to just RMP, indicating that the inhibitors were effective at blocking the mechanism by which RMPs enhance platelet aggregation. This may indicate that RMP affects platelet-platelet interactions and/or platelet aggregate stabilization or may not be effective in patients taking these drugs. However, these experiments were conducted in PRP and not whole blood, negating other mechanisms such as secondary hemostasis. When RMP was tested in the whole blood of a patient with aplastic anemia, the presence of RMP resulted in coagulation [12]. In addition, one limitation of this experiment is that thimerosal was used as a preservative for RMPs, and thimerosal is known to induce platelet aggregation [29]. However, the dose used in our study was 20–40 times lower than what was found to generate platelet aggregation in PRP samples [29].

As RMPs have been found to increase clotting time in several bleeding models, this study provides a clearer picture of how RMPs enhance both primary and secondary hemostatic activity. For primary hemostasis, RMP enhances platelet aggregation in the presence of arachidonic acid, ADP, and collagen and may affect GPIIb/IIIa and P2Y_12_. For secondary hemostasis, RMP is effective in enhancing coagulation in factors VII, VIII, IX, and XI-deficient plasma but not factor XII-deficient plasma. Additional research aimed at a better understanding of MP-mediated hemostasis may lead to new strategies for the safe and effective control of excessive bleeding in surgery, trauma, and other bleeding conditions.

## Electronic Supplementary Material

Below is the link to the electronic supplementary material.

Supplementary Material 1

## Data Availability

The datasets generated during and/or analysed during the current study are available from the corresponding author upon reasonable request.

## Abbreviations

A: velocity of clot formation
ADP: adenosine diphosphate
ANOVA: analysis of variance
AUC: area under the curve
CI: coagulation index
GPIIb/IIIa: glycoprotein IIb/IIIa
K: time to 20 mm amplitude from start of clot formation
MA: maximum strength of clot
MP: microparticles
PPP: platelet-poor plasma
PRP: platelet-rich plasma
R-time: lag time until initial fibrin formation
RBC: red blood cells
RMP: red blood cell-derived microparticles
TEG: thromboelastography
TF: tissue factor
