# Supplementary material for: Red cell microparticles produced using high-pressure extrusion enhance both primary and secondary hemostasis
Source: Pharmacol Rep. 2025 Jan 8;77(2):508–16. doi: 10.1007/s43440-024-00688-0 (PMC11911262; doi:10.1007/s43440-024-00688-0)
Supplement: Supplementary file 1 — Supplementary Material 1 [file 43440_2024_688_MOESM1_ESM.docx]

Supplementary Materials

Figure S1: Dot plot graphs from flow cytometry used to determine the number of RMPs. (A) shows log scale plot of FS vs SS. (B) shows CD235a positive particles, labeled as FL2 (FL1 indicates negative control fluorescence). Particles present in Box F that are CD235a positive are counted as RMPs. FS: forward scatter; RMPs: red blood cell-derived microparticles; SS: side scatter.
